# Does Residential Green and Blue Space Promote Recovery in Psychotic Disorders? A Cross-Sectional Study in the Province of Utrecht, The Netherlands

**DOI:** 10.3390/ijerph15102195

**Published:** 2018-10-08

**Authors:** Susanne Boers, Karin Hagoort, Floortje Scheepers, Marco Helbich

**Affiliations:** 1 Department of Psychiatry, Faculty of Medicine, University Medical Centre Utrecht, Heidelberglaan 100, 3584 CX Utrecht, The Netherlands; susanne.boers@gmail.com (S.B.); f.e.scheepers-2@umcutrecht.nl (F.S.); 2 Department of Human Geography and Spatial Planning, Faculty of Geosciences, Utrecht University, Princetonlaan 8a, 3584 CB Utrecht, The Netherlands; m.helbich@uu.nl

**Keywords:** schizophrenia, psychotic disorders, health data, environmental factors, green space, blue space

## Abstract

Mental health is reportedly influenced by the presence of green and blue space in residential areas, but scientific evidence of a relation to psychotic disorders is scant. We put two hypotheses to the test: first, compared to the general population, psychiatric patients live in neighborhoods with less green and blue space; second, the amount of green and blue space is negatively associated with the duration of hospital admission. The study population consisted of 623 patients with psychotic disorders who had been admitted to the psychiatric ward of an academic hospital in Utrecht, The Netherlands from 2008 to 2016. Recovery was measured by length of stay. Structured patient data was linked to socio-economic status and the amount of green and blue space in the residential area. Associations were assessed by means of regression models controlling for confounding factors. Compared to the general population, psychiatric patients had a significantly lower amount of green space in their neighborhood. This result was not confirmed for blue space. Furthermore, no significant associations were found between green and blue space and the duration of hospital stay. In conclusion, previous studies focusing on other mental disorders, like anxiety or depression, found positive mental health effects of green and blue space in the neighborhood. We were not able to confirm significant effects among our study population on duration of admission, however. Future research focusing on psychotic patients could investigate the influence of exposure to green and blue space on other influences and outcomes on mental health.

## 1. Introduction

A high burden of disease is caused by mental disorders [1], particularly those in the psychotic spectrum, which account for a large share of the disease-adjusted life years attributed to mental disorders [2]. To decrease the burden, we need to understand the factors that influence psychotic disorders and the interaction between these. Besides genetic, biological, psychological, and social factors, which are often associated with psychotic disorders, environmental factors may also be involved [3]. Common aspects of the natural environment near people’s homes are green space (a park or woodland) and blue space (bodies of water). Many studies have examined the relation of green and blue space with general mental health [3,4,5,6,7], but research into the effect of green and blue space on specific mental disorders is scarce. In some experimental studies, exposure to green space was found to improve mental health [8,9,10,11]. In comparison to the built environment [12], green space is thought to improve social cohesion [13], reduce stress, facilitate restoration of attention problems and fatigue, improve mood [14], and reduce symptoms of major depression [14,15,16]. Exposure to blue space has been found to reduce stress [17]. Several cross-sectional studies have revealed a negative association between green space, depression [18], suicide mortality [19], and stress [20] but a positive association between time spent in green space and mental health improvement [21]. A recent systematic review [6] described 28 studies that had investigated the possible benefit of green and blue space on mental health. The findings were inconsistent, however. The reviewer cited the limited number of studies that were included and the heterogeneity across the studies regarding the assessment of green and blue space. Thus far, little research has dealt with the influence of green or blue space on psychotic disorders.

This study addresses that gap in the literature by investigating the possible protective effect of living near green or blue space among patients with diagnosed psychotic disorders in the province of Utrecht, The Netherlands. Specifically, it assesses the relation between green space or blue space in the residential environment of patients that have been diagnosed with a psychotic disorder and admitted to an academic hospital. We hypothesized that psychiatric patients live in neighborhoods where the absence of green or blue space is more pronounced than in areas occupied by the general population. Then we tested whether the availability of green or blue space is negatively associated with the duration of admission (as a measure of recovery) for the psychotic disorder.

## 2. Materials and Methods 

### 2.1. Study Population 

In 2013, the Province of Utrecht had 640,610 addresses and 1,245,294 inhabitants [22], of whom 5056 (0.4%) had a diagnosis of ‘schizophrenia or any other psychotic disorder’ [23]. The study population consisted of patients with a psychotic disorder who had been admitted to the inpatient department of psychiatry, University Medical Center (UMC) Utrecht, between January 2008 and June 2016. After selecting patients whose primary diagnosis was a psychotic disorder, we filtered out those living outside the province based on their residential address. This resulted in the inclusion of 48% of all patients. If one patient had multiple treatments within the study period, we used data from the latest admission because it was up to date. In total, 623 patients were included in the analysis. Approval for the study was obtained from the medical ethics review committee.

### 2.2. Health Data

The health data originate from the UMC Utrecht clinical databases. To guarantee privacy, data from patients who used the inpatient services of the psychiatric department of the UMC Utrecht were anonymized and stored in the UMC Utrecht research database for analysis. We selected patients with the diagnosis ‘schizophrenia spectrum and other psychotic disorders’ according to the Diagnostic and Statistical Manual of Mental Disorders (DSM-IV). As dependent variables for subsequent regression models, we operationalized the length of stay as a measure of severity/recovery. The length of stay refers to the number of days for which patients had been admitted to the psychiatric ward at the UMC Utrecht, so the duration could only be calculated for inpatients.

This study was approved by the medical ethics committee of the UMC Utrecht (reference number WAG/nt/16/033895).

### 2.3. Measures of Green and Blue Space 

The key explanatory variables were the availability of green and the availability of blue space, both given in percentages, near the patient’s home. Green space refers to agricultural areas, natural areas, or artificially installed greenery; blue space refers to fresh water or salt water bodies. Both green and blue space data were extracted from the most recent Dutch land use database for 2012 with a spatial resolution of 25 × 25 m per raster cell [24]. This land use database differentiates 43 land use types, which were reclassified and aggregated using the ArcGIS 10.4 software (ESRI, Redlands, CA, USA) as follows: green space comprises agricultural areas (categories 1–6, 9), forests (categories 11–12), natural areas (categories 30–43, 45, and 61–62); blue space comprises fresh water and salt water (categories 16 and 17). In accordance with prior studies [6,25], we considered the amount of green and blue space within a circular buffer of 300 m centered on a patient’s home address (in %). 

### 2.4. Covariates

Besides environmental exposure, the analyses considered both individual and area-based covariates. Covariates on an individual level were gender and age (in years). Covariates based on area were urbanicity and socio-economic status. Urbanicity adjusts for urban-rural differences in mental health [26] and is operationalized as address density. Addresses were extracted from the Dutch cadaster for the year 2016 and we computed the number of address locations within a 300-m buffer around the patients’ home location. Socio-economic status of the neighborhood is represented by means of average residential property value (in 1000 Euros). This variable was collected from Statistics Netherlands on a 100-m grid for the year 2012. 

### 2.5. Statistical Analyses

We used descriptive statistics to summarize the data. To test the hypothesis that psychiatric patients live in neighborhoods with less green and blue space compared to the general population, we performed a series of Chi^2^ tests. We carried out non-parametric Spearman correlations between the variables to gain insight into the bivariate associations and to identify problems due to multicollinearity. We selected the Spearman correlation coefficient because our data did not exactly follow a normal distribution. To study the extent to which natural environments (green and blue space) are associated with the severity of symptoms of psychotic patients, we performed a multivariate regression analysis by means of fitting ordinary least squares regression models. We regressed the dependent variable ‘length of stay’ on blue space and green space while adjusting for the covariates including gender, age, socio-economic status and urbanicity. We estimated two different models with increasing complexity. Model 1 included green and blue space and was adjusted for gender and age. Model 2 additionally considered urbanicity and area-based socio-economic status. SPSS 22.0 (SPSS Inc., Chicago, IL, USA) was used for the data preparation and statistical analysis.

## 3. Results

### 3.1. Descriptive Statistics of the Study Population 

Our study population consisted of 623 patients, of whom 70% were male (Table 1). The mean age was 38 years, ranging from 11 to 94 years. The patient records included visits between 2008 and 2016 whereas the average length of stay was 40 days and varied widely between 1 and 361 days. About 50% of the patients were admitted for a period shorter than a month. The mean availability of green and blue space in the residential environment is relatively low, and with 5.6% green and 4.2% blue, greenery and water are almost equally available. Both distributions were slightly right-skewed, especially for green space. Approximately 65% of the patients had no green space near their home. The average residential property value was 238,000 Euros. However, the residential property value varied between 90,000 Euros and 1,120,000 Euros. The mean number of addresses was 1085. The standard deviation, at 548, was high.

### 3.2. Bivariate Analyses 

Table 2 shows the bivariate associations between the variables. Green space and blue space were not significantly correlated with the length of stay, however, confirming the expected direction. Older people had a shorter duration of admission. Chi^2^ tests showed that psychiatric patients live significantly more often in neighbourhoods with no green space than the general population of the province of Utrecht (Chi^2^ = 44.770; *p*-value < 0.010). This result was not confirmed for blue space. The proportion of psychiatric patients with no blue space within their residential area did not significantly differ from the general population (Chi^2^ = 0.147; *p*-value = 0.701).

### 3.3. Regression

The *F*-test of the partially adjusted regression model 1 (*F* = 4.588; *p* = 0.001) and the fully adjusted model 2 (*F* = 3.057; *p* = 0.006) showed statistical significance (Table 3). Both adjusted *R*^2^ are, at 3%, low. Independent of the fitted model, we found no statistical evidence that either green space or blue space is correlated with our response variable, namely length of stay of patients diagnosed with schizophrenia and other psychotic disorders. Because of a skewed distribution of green and blue space, both variables were also log transformed and grouped. However, these approaches did not change our results; both green and blue space remained insignificant. Similarly, urbanicity and socio-economic status were not related with the response variable. Only age, consistently across both models, was negatively related and statistically significant.

## 4. Discussion 

While the link between natural environment, particularly green space, and health is stressed in numerous studies with various study designs, less is known about the beneficial effects of blue space. Although previous studies focused on green space and mental health [3,4,5,6,7], little research is dedicated to patients with a psychotic disorder and how exposure to both green space and blue space in the residential area would affect people’s length of stay in mental health institutions or hospitals. 

This is the first study, to our knowledge, investigating the associations between green space and blue space in patients diagnosed with schizophrenia or another psychotic disorder. Our hypothesis that patients with a psychotic disorder reside in neighborhoods with less green and blue space availability compared to the general population was partly confirmed. While we found that psychiatric patients live in areas with less green space, no evidence was found for blue space. Moreover, our regression models did not confirm that green and blue space availability is negatively associated with the duration of hospital admission. Three explanations are plausible to explain the latter result. First, it might be that length of stay in a hospital is a less appropriate outcome measure to unravel the relation between the natural environment and psychotic disorders. One could argue that the natural environment where people live would not be protective during hospital stays. Apparently, according to our results, more green and blue space availability does not result in a shorter admission period. Conceivably, the length of stay does not give a valid indication of the extent of recovery. It should be kept in mind that other studies investigated less severe, self-reported mental health outcomes [26], such as depression symptoms or the quality of life, and not clinical data. 

A second possible explanation is that the radius of 300 m that we set for this study was too small, though it proved to be a valid distance to represent the immediate surroundings [6]. Other Dutch studies have drawn a larger radius, for example 1000 or 3000 m. A study assessing the association between green and blue space availability on anxiety, mood and substance use disorder found a significant influence of green space availability in a 1000-meter radius for anxiety disorder but not for any other disorder, nor for blue space availability [26]. A study on the influence of either grass or tree greenness found only positive results at a radius of 1000 m on self-reported health and no effects at 300 m [27].

A third reason could be that the effect of the natural environment on the status of psychotic disorders is less pronounced than it is on other psychiatric disorders. Most studies on mental health and the natural environment have included people with anxiety or mood disorders [6], hypothesizing that the natural environment decreases stress and increases exercise time, thereby reducing symptoms of anxiety or depression. 

Several limitations should be considered when interpreting our results. First, though useful for hypothesis generation, the research design is cross-sectional. We recommend that future studies be longitudinal. Second, although we controlled for demographics and socio-economic status, our models remained unadjusted for other factors (e.g., lifestyle, physical activity) known to affect mental health. Third, our sample showed limited variance concerning the availability of green and blue space for inhabitants of Utrecht as well as for patients with schizophrenia and psychotic disorders. Further, the skewness in these two environmental variables made them less powerful as predictors in the analyses. The amounts of green and blue space are calculated around a patient’s home address that was registered by the hospital. But as argued elsewhere [3], people spend a limited amount of time at home and are exposed to other environments along their daily trajectories or over the course of their life. Fourth, limited due to data availability, SES was only represented through the proxy variable property value. Other variables such as homeownership may be more suitable. 

In view of the mixed findings we suggest three ways to pursue more reliable insight into the interaction between green and blue space availability and psychotic disorders. First, the majority of studies have focused on the natural environment, either in the neighborhood or as operationalized through buffers, rather than using nature exposure during the day (e.g., along walks and during activities or at the workplace). Advances in geotechnology and GPS make it possible to refine exposure assessments in future research. Similarly, historical data on natural environmental exposure has rarely been incorporated in research, although the influence of exposure over the life course might be of great interest [3,28].

## 5. Conclusions

This study explored how natural environments may be associated with psychotic disorders. Our results did not support the increasingly reported mental health benefit of green and blue space. Across our regression models, the associations tuned out to be insignificant, though the direction of the effect was in line with our hypothesis. Nonetheless, this study does contribute to the literature by exploring the relationship between environments and psychotic disorders by analyzing clinical data, which so far has rarely been attempted.

## Figures and Tables

**Table 1:** Descriptive statistics of the study population.

Variable	Range	Mean	SD
Length of stay (days)	1–361	40	38.3
Green space (300 m)	0–94	5,6	14.4
Blue space (300 m)	0–46	4,2	5.0
Gender (female, male)	30%; 70%		
Age (years)	11–94	38	14
Socio-economic status (SES) (in 1000 €)	90–1120	228	102
Address density (300 m)	5–2677	1085	548

**Table 2:** Spearman correlation coefficients.

		Green Space	Blue Space	Gender	Age	SES	Urbanicity
Length of stay	Correlation	0.018	0.023	−0.031	−0.200	0.026	−0.010
	*p*-value	0.653	0.577	0.441	0.000	0.542	0.808
Green space	Correlation	1	−0.125	−0.050	−0.081	0.140	−0.574
	*p*-value		0.002	0.217	0.045	0.001	0.000
Blue space	Correlation	−0.125	1	0.030	−0.009	0.078	0.004
	*p*-value	0.002		0.460	0.818	0.070	0.913
Gender	Correlation	−0.050	0.030	1	0.189	0.042	−0.018
	*p*-value	0.217	0.460		0.000	0.315	0.654
Age	Correlation	−0.081	−0.009	0.189	1	−0.106	0.093
	*p*-value	0.045	0.818	0.000		0.011	0.019
SES	Correlation	0.140	0.078	0.042	−0.106	1	−0.328
	*p*-value	0.001	0.070	0.315	0.011		0.000
Urbanicity	Correlation	−0.574	0.004	−0.018	0.093	−0.328	1
	*p*-value	0.000	0.913	0.654	0.019	0.000	

**Table 3:** Regression results.

Model		Coef. Unstandardized	Std. Error	Coef. Standardized	*t*-Values	*p*-Values
1	Intercept	55.653	4.960		11.220	0.000
	Green space	0.038	0.163	0.010	0.232	0.817
	Blue space	0.381	0.343	0.047	1.113	0.266
	Male	0.503	3.578	0.006	0.141	0.888
	Age	−0.466	0.118	−0.173	−3.951	0.000
2	Intercept	53.002	8.100		6.544	0.000
	Green space	0.059	0.185	0.015	0.321	0.748
	Blue space	0.392	0.346	0.049	1.133	0.258
	Male	0.546	3.596	0.007	0.152	0.879
	Age	−0.468	0.119	−0.174	−3.934	0.000
	SES	0.005	0.017	0.012	0.278	0.781
	Urbanicity	0.001	0.004	0.018	0.371	0.711
